# Different Endophytes Colonized in Various Lotus Root Varieties and Their Associated Mealy and Crunchy Properties

**DOI:** 10.3390/ijms26104529

**Published:** 2025-05-09

**Authors:** Yufei Wei, Xinyan Zhou, Meiping Gao, Yangxiu Ou, Yifeng Hu, Wen Jiang, Huiping Jiang, Shangdong Yang

**Affiliations:** 1 Guangxi Key Laboratory of Agro-Environment and Agro-Products Safety, National Demonstration Center for Experimental Plant Science Education Guangxi Agricultural College, Guangxi University, Nanning 530004, China; wyf18177217289@163.com (Y.W.); zhouxinyan0923@163.com (X.Z.); 2 Vegetable Research Institute, Guangxi Academy of Agricultural Sciences, Nanning 530007, China; gmp2009@163.com (M.G.); oyx1322@139.com (Y.O.); sunmoonfeng@126.com (Y.H.); jiangwen5476581@163.com (W.J.)

**Keywords:** lotus root, endophytic microbial compositions, metabolome, polysaccharides, texture variation

## Abstract

Lotus root texture significantly influences consumer preferences and market value, yet the role of endophytes in determining the distinct mealy (ML) and crunchy (CL) textural properties remains unclear. This study aimed to clarify the relationship between endophyte composition and metabolic characteristics underlying the texture differences between ML and CL lotus root varieties. Two lotus root varieties (ML and CL) were analyzed for endophytic microbial communities using high-throughput sequencing methods. Metabolite profiling of cellulose, starch, pectin, soluble sugars, and proteins was conducted using standard biochemical assays. The findings revealed higher cellulose, starch, and pectin content in mealy lotus root (ML) varieties than those in crunchy lotus root (CL) varieties. Additionally, the functions of cellulose-degrading and protein-producing microorganisms, such as Firmicutes, Bacteroides, *Exiguobacterium*, *Bradyrhizobium*, and *Basidiomycota,* were primarily enriched in the ML varieties. In contrast, the CL varieties had specific dominant endophytic bacterial genera, such as *Myxococcota*, *Geobacter*, *Paludibacteraceae*, *Rhodocyclaceae*, *Comamonadaceae*, *Micromonosporaceae*, *Sideroxydans*, *Bacillus*, *Lactococcus*, *Oxalobacteraceae*, and *Treponema*. These results indicate that different endophytes are associated with the development of mealy and crunchy properties. Understanding these microbial–metabolic relationships offers practical implications for selective breeding and agricultural management aimed at texture improvement. Future research should elucidate the specific metabolic pathways regulated by these endophytes to facilitate targeted agricultural interventions.

## 1. Introduction

Lotus (*Nelumbo nuciferais* Gaertn.) has been cultivated as a significant aquatic vegetable in China and India for over 7000 years [1]. Lotus is mainly cultivated in China in the upper and lower reaches of the Yangtze River, and is widely cultivated in southern China. Lotus roots are rich in starch, sugar, protein, minerals, and other nutrients, making them a popular health food [2]. It is rich in various bioactive ingredients and antioxidants, such as alkaloids, glycosides, flavonoids, triterpenoids, vitamins, phenols, tannins, and flavonoids, which contribute to immune regulation, and antioxidant, anti-diabetes, and anti-obesity activities [3,4]. Polysaccharides and phenolic compounds are the primary bioactive components [4]. However, different lotus root varieties exhibit significant differences in the polysaccharide content, composition, and structure [5].

The cell wall is primarily composed of pectin, cellulose, hemicellulose, structural glycoproteins, phenolic esters, minerals, and enzymes. Among them, cellulose—a stable polysaccharide—serves as the primary structural framework of the cell wall, providing rigidity and resistance, whereas hemicellulose provides plasticity and tensile strength. The cell wall integrity is crucial for lotus root texture [6]. Starch, composed of glucose polysaccharides, is the most significant component in lotus root, and consists of amylopectin and amylose linkages [7]. Generally, the average starch content in lotus roots is 10–20% of its fresh weight and varies across different lotus root varieties. Based on the lotus root texture, it can be classified into brittle (higher water and sugar content and lower starch content) and powdery (high starch and low moisture content). Different varieties of lotus roots exhibit significant differences in starch content and amylose/amylopectin ratio, owing to the growth environment, cultivation methods, and genetic factors. Additionally, root textures are associated with variations in protein, soluble sugar, vitamin C, cellulose, hemicellulose, and phenol content [8,9].

Additionally, endophytes that inhabit the entire plant can positively affect plant growth [10] and enhance plant stress tolerance by producing biological agents [11,12]. Plant species, organ types, and phenological stages significantly affect the endophytic flora of plants [13,14,15]. Endophytic bacteria enhance plant growth through nitrogen fixation, production of indoleacetic acid and iron carriers, and enhancement of stress resistance [16,17]. Endophytic fungi can produce numerous secondary metabolites, such as alkaloids, phenols, terpenoids, and other structural types in plants [17] and facilitate plant growth [18,19]. Additionally, plant tissues and growth conditions alter the structure of endophytic bacteria [20].

Research on lotus roots has primarily focused on postharvest preservation [21], antioxidation [22], and extract function [23]. However, there are limited studies on the role of microorganisms in the quality of lotus roots. This study aimed to assess the differences in the endophytic microbial community structure between mealy (ML) and crunchy lotus root (CL) varieties and to explore whether these microbes contribute to the formation of ML and CL properties.

## 2. Results

### 2.1. Diversity of Endophytic Microorganisms in Lotus Root

As presented in Table 1, the moisture content in the CL varieties (the same below) was significantly higher than that in the ML varieties, whereas starch content was significantly lower than that in the ML varieties (the same below). However, there were no significant differences in protein and soluble sugar contents between the CL and ML varieties.

**Figure 1:**
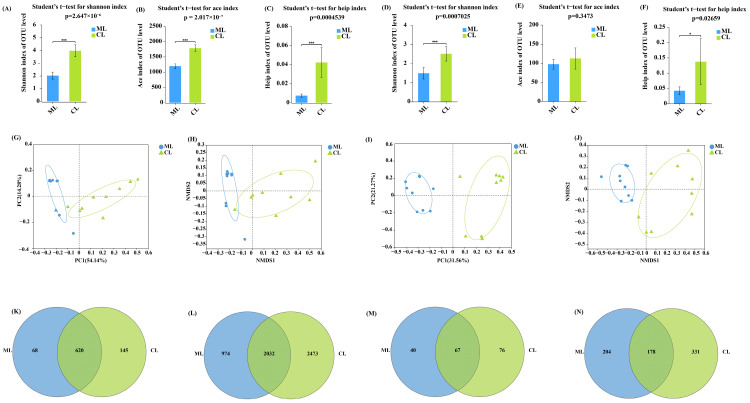
Comparison of endophytic microbial community structures at a 97% similarity level between ML and CL Varieties (OTU level). (**A**) The Shannon index indicates endophytic bacterial diversity. (**B**) The ace index indicates endophytic bacterial richness. (**C**) The Heip index indicates endophytic bacterial evenness. (**D**) The Shannon index indicates endophytic fungal diversity. (**E**) The ace index indicates endophytic fungal richness. (**F**) The Heip index indicates endophytic fungal evenness. (**G**) PCoA of endophytic bacterial communities. (**H**) NMDS of endophytic bacterial communities. (**I**) PCoA of endophytic fungal communities. (**J**) NMDS score plot of endophytic fungal communities. (**K**) Venn diagram analyses of endophytic bacteria at the genus level. (**L**) Venn diagram analyses of endophytic bacteria at the OTU level. (**M**) Venn diagram analyses of endophytic fungi at the genus level. (**N**) Venn diagram analyses of endophytic fungi at the OTU level. ML: mealy lotus varieties; CL: crunchy lotus varieties; PCoA: principal coordinate analysis; NMDS: non-metric multidimensional scaling; OTU: operational taxonomic unit. *p* values are indicated by asterisks: * *p* < 0.05; *** *p* < 0.001.

As illustrated in Figure 1A–F, the diversities of endophytic bacteria and fungi in the CL varieties were significantly higher than those in the ML varieties. However, only the bacterial richness in the CL varieties was significantly higher than that in the ML varieties, and there was no significant difference in fungal richness between the CL and ML varieties. Additionally, the results of unweighted principal coordinate analysis and non-metric multidimensional scaling indicated that significant differences in endophytic microbial compositions can be detected between the ML and CL varieties (Figure 1G–J). Moreover, at the genus and operational taxonomic unit levels, more abundant unique endophytic microbes were observed in the CL varieties than those in the ML varieties (Figure 1K–N).

The results revealed that higher microbial diversity and more abundant unique endophytic microbes can be detected in the CL varieties than in the ML varieties. Additionally, they indicated that diverse lotus qualities recruited different endophytic microorganisms to the roots.

### 2.2. Composition of Endophytic Microorganisms in Lotus Root

**Figure 2:**
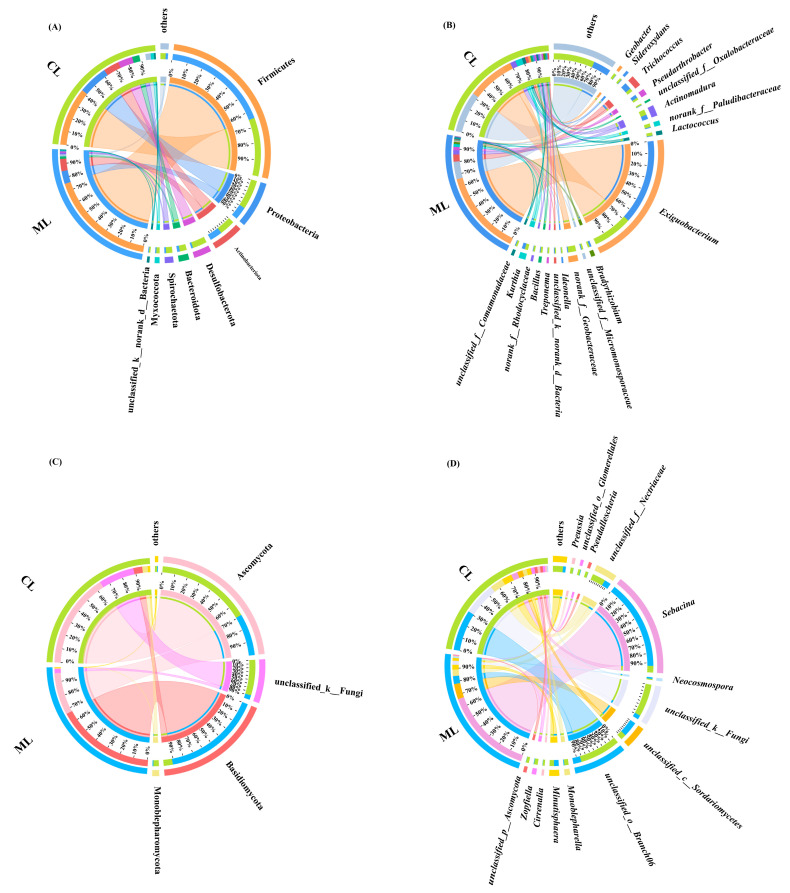
(**A**) Compositions of endophytic bacterial communities at the phylum level; (**B**) compositions of endophytic bacterial communities at the genus level; (**C**) compositions of endophytic fungal communities at the phylum level; (**D**) compositions of endophytic fungal communities at the genus level. ML: mealy lotus varieties, CL: crunchy lotus varieties.

Additionally, the dominant endophytic bacterial phyla in the ML and CL varieties were Firmicutes, Proteobacteria, Actinobacteriota, Bacteroidota, Spirochaeta, and Desulfobacterota. However, Myxococcota was more dominant in the CL varieties than in the ML varieties (Figure 2A).

At the genus level, the top five dominant endophytic bacterial genera in the ML varieties were *Exiguobacterium* (66.43%), *Trichococcus* (5.57%), *Actinomadura* (3.31%), *Pseudarthrobacter* (2.64%), and *Kurthia* (2.32%). In contrast, the top five dominant endophytic bacterial genera in the CL varieties were *Exiguobacterium* (30.93%), *Actinomadura* (3.44%), *Lactococcus* (2.44%), *Paludibacteraceae* (2.80%), and *Kurthia* (2.47%). Additionally, *Geobacter*, *Paludibacteraceae*, *Rhodocyclaceae*, *Comamonadaceae*, *Micromonosporaceae*, *Sideroxydans*, *Bacillus*, *Lactococcus*, *Oxalobacteraceae*, and *Treponema* were uniquely dominant in the CL varieties (Figure 2B).

Moreover, at the phylum level, Basidiomycota, Ascomycota, and unclassified_k_Fungi were the dominant endophytic fungi in the ML and CL varieties; however, their proportions differed between the ML and CL varieties (Figure 2C). At the genus level, *Preussia* and *Pseudallescheria* were unique in the CL varieties as compared to the ML varieties (Figure 2D).

**Figure 3:**
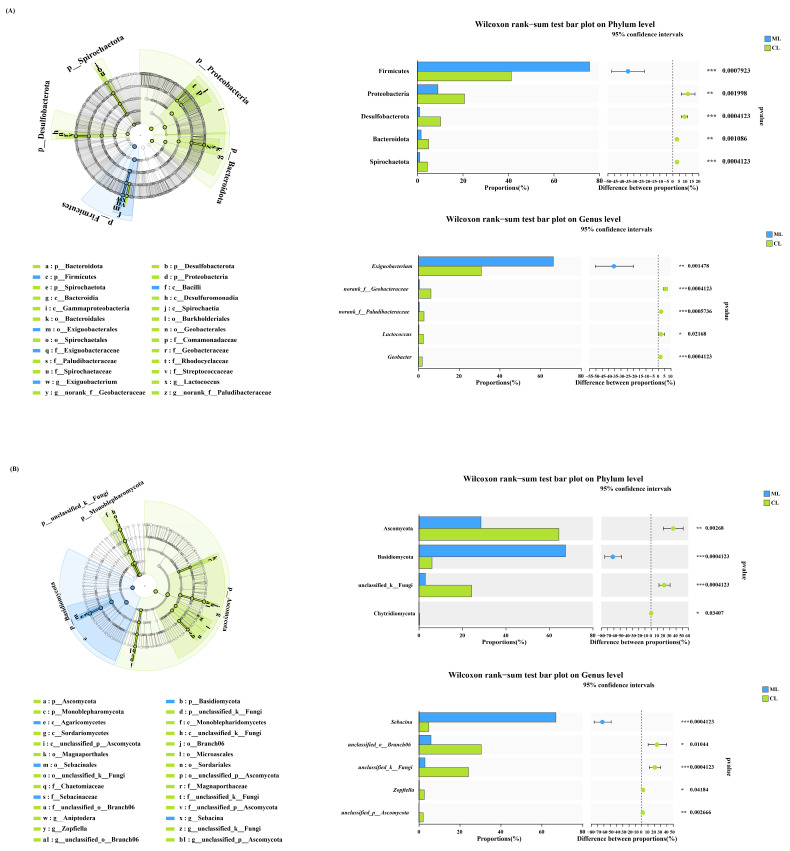
Linear discriminant analysis Effect Size analysis of endophytic bacteria (**A**) and fungi (**B**). Wilcoxon rank-sum test of endophytic bacteria and fungi in lotus root. ML: mealy lotus varieties, CL: crunchy lotus varieties. *p* values are indicated by asterisks: * *p* < 0.05; ** *p* < 0.01; *** *p* < 0.001.

Additionally, the results of linear discriminant analysis Effect Size analysis and Wilcoxon rank sum test for the top five demonstrated that Firmicutes and *Exiguobacterium* were the dominant endophytic bacterial phylum and genus in the ML varieties, with their proportions significantly higher than those in the CL varieties. In contrast, Bacteroidota, Desulfobacterota, Proteobacteria, Spirochaetota, *Geobacter*, and *Lactococcus* were the dominant endophytic bacterial phyla and genera in the CL varieties, with their proportions significantly higher than those in the ML varieties (Figure 3A).

Moreover, Basidiomycota and *Sebacina* were the dominant endophytic fungal phylum and genus in the ML varieties. In contrast, Ascomycota, Monoblepharomycota, unclassified_k__Fungi, Aniptodera, *Zopfiella*, and unclassified_k__Fungi were the dominant endophytic fungal phyla and genera in the CL varieties. The proportions of Basidiomycota and *Sebacina* in the ML varieties were significantly higher than those in the CL varieties, whereas the proportions of Ascomycota, unclassified_o__Branch06, unclassified_k__Fungi, and *Zopfiella* in the CL varieties were significantly higher than those in the ML varieties (Figure 3B).

**Figure 4:**
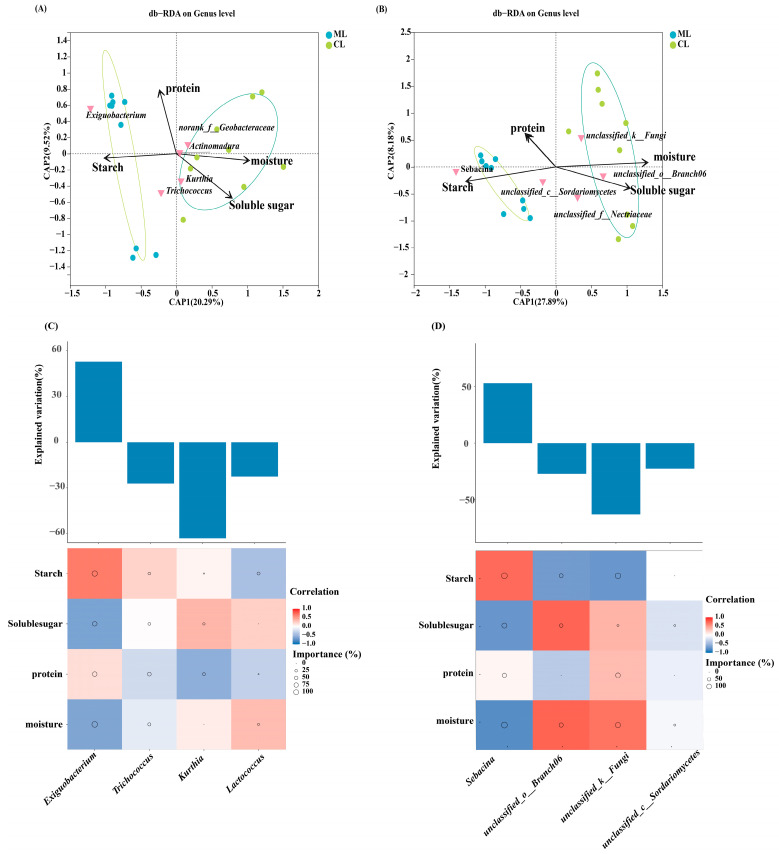
Distance-based redundancy analysis, a statistical method for assessing biological traits and bacterial (**A**) and fungal (**B**). Biological traits for relative abundance and differences of bacteria (**C**) and fungi (**D**) based on correlation and optimal multiple regression model. The primary predictors are identified. The size of the circle represents the significance of the variable (the proportion of explained variability calculated using the multiple regression model and variance decomposition analysis). Colors represent Spearman correlations. ML: mealy lotus varieties, CL: crunchy lotus varieties.

Distance-based redundancy analysis was used to assess the relationships between endophytic microbial genera and quality indices in the ML and CL varieties. The analysis revealed that protein content was positively correlated with *Exiguobacterium*, water content was positively correlated with *Actinomadura* and *Kurthia*, and soluble sugar content was positively correlated with *Kurthia* and *Trichococcus* (Figure 4A,B). Additionally, the results of random forest variable importance analysis revealed that *Exiguobacterium* was significantly positively correlated with soluble sugar content, whereas *Trichococcus* was negatively correlated with starch and protein content.

Additionally, the fungal genera, such as *Sebacina*, were negatively correlated with starch content but positively correlated with soluble sugar content. Moreover, unclassified_c_Sordariomycetes and unclassified_f_Nectriaceae were positively correlated with the soluble sugar content (Figure 4C,D).

Firmicutes, *Exiguobacterium*, and *Bradyrhizobium* were the dominant endogenous bacterial phyla and genera, respectively, in the ML varieties, with significantly higher abundances than those in the CL varieties. The abundance of Bacteroides was significantly higher in the CL varieties than that in the ML varieties. *Preussia* and *Pseudallescheria* are the dominant endogenous bacterial genera in the CL varieties. In contrast, the abundance of Bacteroidota was significantly higher in the CL varieties than that in the ML varieties.

**Figure 5:**
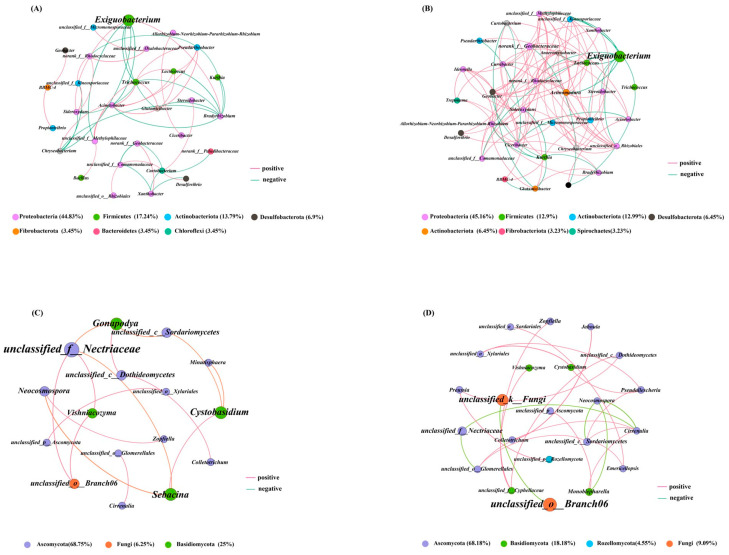
Microbial colinear network of different quality lotus roots. (**A**,**B**) bacteria; (**C**,**D**) fungi; (**A**,**C**) ML; (**B**,**D**) CL. ML: mealy lotus varieties, CL: crunchy lotus varieties.

As illustrated in Figure 5A, the highest proportion of endophytic bacterial genera in the ML varieties, *Exiguobacterium*, was negatively correlated with Proteobacteria, Firmicutes, and Actinobacteria. In contrast, although *Exiguobacterium* in the CL varieties exhibited a significant negative correlation with other endophytic bacteria, it exhibited a more significant positive correlation with the other endophytic bacteria (Figure 5B).

In the ML varieties, unclassified_f_Nectriaceae and *Sebacina* were positively correlated with the other endophytic fungi (Figure 5C). In contrast, the CL varieties had a more complicated network structure of endophytic fungi. For example, the primary nodes of unclassified_k_Fungi and unclassified_o_Branch06 were positively and negatively correlated, respectively, with other endophytic fungi. (Figure 5D). This indicates that the CL varieties required more intricate microbial networks of endophytic microbes than those of the ML varieties.

### 2.3. Function Prediction of Endophytic Microorganisms in Lotus Roots

**Figure 6:**
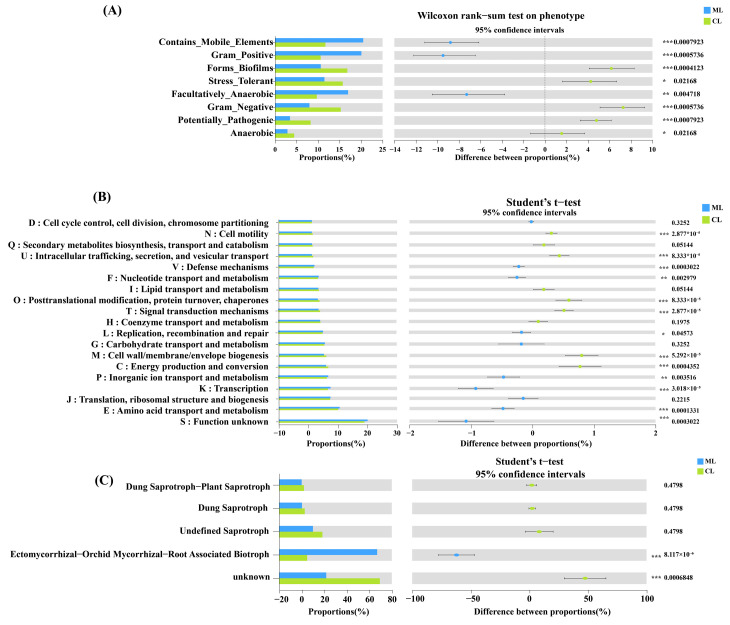
(**A**) BugBase predicted the phenotype shift of the endophytic bacterial community between ML and CL varieties. (**B**) Endophytic bacterial community phenotypes from ML and CL varieties are identified using Clusters of Orthologous Groups analysis and (**C**) fungal functional groups based on operational taxonomic units. Wilcoxon rank-sum test and Student’s *t*-test of endophytic bacteria (**A**) and fungi (**B**) in lotus root. ML: mealy lotus varieties, CL: crunchy lotus varieties. *, 0.01 ≤ *p* ≤ 0.05; **, 0.001 ≤ *p* ≤ 0.01; and ***, *p* ≤ 0.001.

The results of BugBase analysis revealed that nine groups of endophytic bacteria can be divided between the ML and CL varieties (Figure 6A). Among them, the proportions of contains_mobile_elements, gram_positive, and facultative anaerobic bacteria in ML varieties were significantly higher than those in CL varieties. In contrast, the proportions of forms_biofilms, stress_tolerant, gram_negative, potentially_pathogenic, and anaerobic bacteria in the CL varieties were significantly higher than those in ML varieties. Based on the KEGG database, in the ML varieties, the endophytic bacterial community functions, such as inorganic ion transport and metabolism, replication, recombination, and repair, transcription, nucleotide transport and metabolism, and amino acid transport and metabolism were significantly higher than those in CL varieties (Figure 6B). In contrast, in the CL varieties, the defense mechanism, intracellular trafficking, secretion and vesicular transport, signal transduction mechanisms, posttranslational modification, protein turnover, and chaperones, cell motility, cell wall/membrane/envelope biogenesis, and energy production and conversion were significantly higher than those in ML varieties.

Additionally, the results of FUNGuild analysis revealed that the dominant groups among the ML and CL varieties included dung saprotroph–plant saprotroph, dung saprotrophs, undefined saprotrophs, and ectomycorrhizal-orchid mycorrhizal-root-associated biotrophs (Figure 6C). Specifically, the ectomycorrhizal-orchid mycorrhizal-root-associated biotrophs in the ML varieties were significantly higher than those in the CL varieties.

These findings suggest that CL varieties harbor a more functionally diverse endophytic microbial community than ML varieties.

### 2.4. Composition of Metabolites in Lotus Root

**Figure 7:**
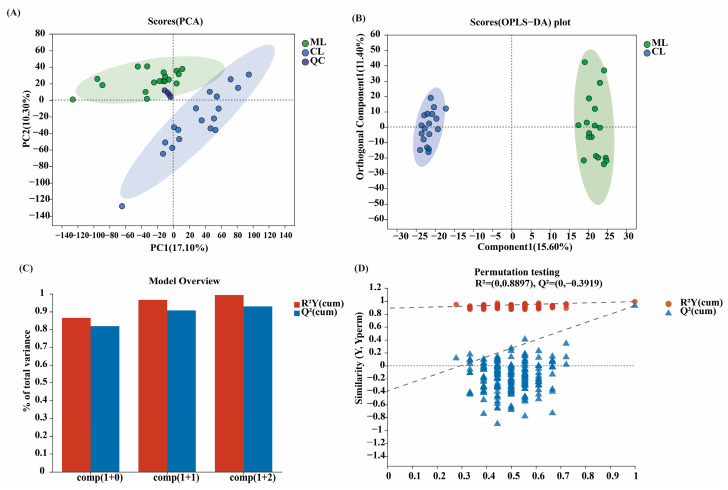
(**A**) PCA analysis of root metabolites using liquid chromatography–mass spectrometry ESI (+) between ML and CL varieties. (**B**) Score plots of orthogonal partial least squares discriminant analysis of ML and CL metabolites. (**C**) Principal component number analysis. (**D**) Model validation. PCA: principal component analysis; ESI (+): positive ion mode; ML: mealy lotus varieties; CL: crunchy lotus varieties.

For the results of principal component analysis and orthogonal partial least square discriminant analysis (R^2^ = 0.8897 and Q^2^ = −0.3919), significant differences in metabolites can be detected, indicating that significant differences in metabolites can be detected between the ML and CL varieties (Figure 7A–D).

**Figure 8:**
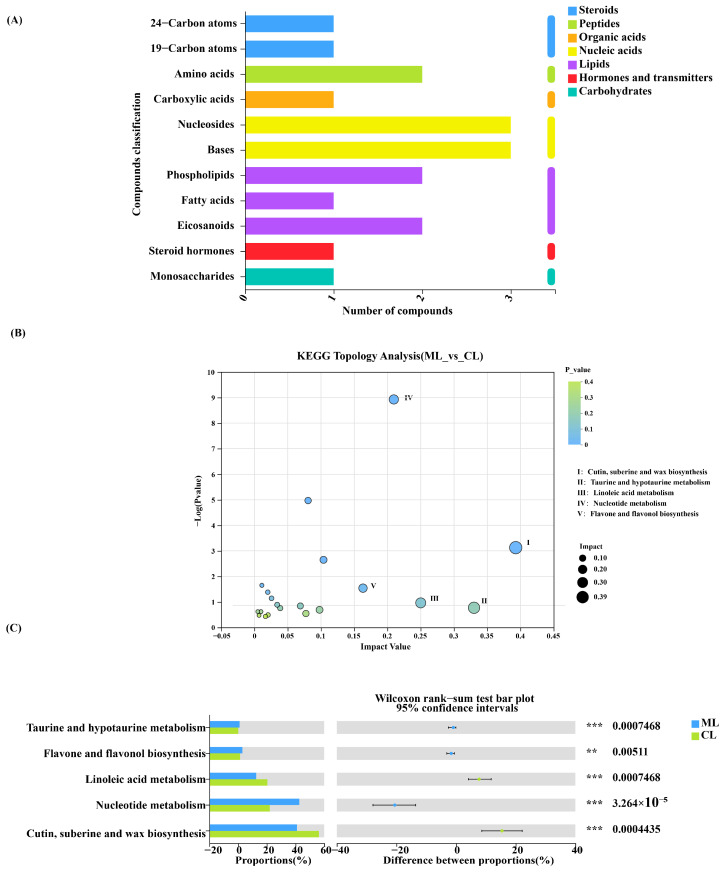
(**A**) KEGG pathway classification: metabolites detected and annotated. (**B**) Metabolic pathway enrichment study of differentially abundant metabolites between ML and CL root metabolites. (**C**) The alterations in root exudates in various metabolic pathways using KEGG. * The representation is significant. KEGG: Kyoto Encyclopedia of Genes and Genomes; ML: mealy lotus varieties; CL: crunchy lotus varieties. *p* values are indicated by asterisks: ** *p* < 0.01; *** *p* < 0.001.

Based on KEGG topology analysis, carbohydrates, hormones and transmitters, lipids, nucleic acids, organic acids, peptides, and steroids were the primary metabolites between the ML and CL varieties (Figure 8A). Additionally, taurine and hypotaurine metabolism and flavone and flavonol biosynthesis in the ML varieties were significantly higher than those in the CL varieties. In contrast, linoleic acid and nucleotide metabolism and cutin, suberin, and wax biosynthesis in the CL varieties were significantly higher than those in the ML varieties (Figure 8B,C).

Additionally, 31 of the 36 lotus root metabolites in the ML varieties were significantly higher than those in the CL varieties. These included two phenylpropanoids and polyketides, one organoheterocyclic compound, twenty-four lipids and lipid-like molecules, two lignans, neolignans, and related compounds, and one benzenoid compound (Table 2).

### 2.5. Comparison of Metabolites and Biological Characteristics of Endophytic Microbiota in Lotus Root

**Figure 9:**
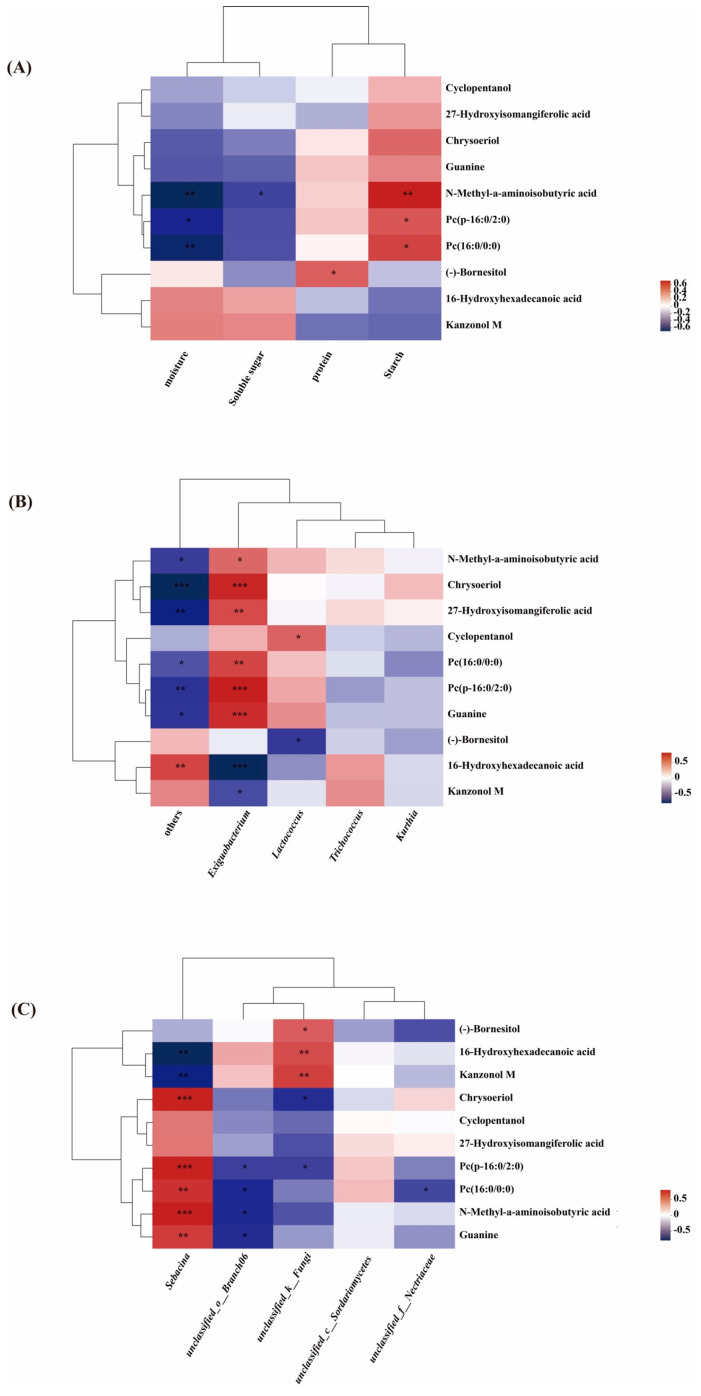
(**A**) Correlation between metabolites and biological characteristics in ML and CL lotus root. (**B**) Correlation between metabolites and endophytic bacterial community in ML and CL lotus root. (**C**) Correlation between metabolites and endophytic fungal community in ML and CL lotus root. Heatmaps analyzed using Spearman’s correlation coefficient (rho value) and *p*-value of metabolites and flora. * 0.01 < *p* < 0.05, ** 0.001 < *p* < 0.01, and *** *p* < 0.001 indicate significant differences. The clustering at the top and left of the graph indicate the metabolites of bacterial groups and the results of hierarchical clustering based on Euclidean distance, respectively. ML: mealy lotus varieties; CL: crunchy lotus varieties.

Based on the heat map analysis, we identified the following—Pc (p-16:0/2:0), *Lactococcus*, and 27-hydroxyisomangiferolic acid were significantly positively correlated with starch, cyclopentanol, and *Exiguobacterium*, respectively. Chrysoeriol was significantly positively correlated with *Exiguobacterium* and *Sarcina* and negatively correlated with unclassified_k__Fungi. Guanine was significantly positively correlated with *Exiguobacterium* and *Sebacina*, and significantly negatively correlated with unclassified_o__branch06. (-)-Bornesitol was significantly positively correlated with protein and unclassified_k__Fungi and negatively correlated with *Lactococcus*. 16-Hydroxyhexadecanoic acid was significantly positively correlated with unclassified_k__Fungi and negatively correlated with *Exiguobacterium* and *Sebacina*. Additionally, kanzonol M was significantly positively correlated with unclassified_k__Fungi and negatively correlated with *Sebacina* and *Exiguobacterium* (Figure 9A–C). In this study, *Exiguobacterium* and *Sebacina* were the dominant endophytic bacterial genera in the ML varieties, indicating an association between the endophytic bacteria and lotus root quality.

## 3. Discussion

Lotus root is rich in flavonoids, vitamins, minerals, and starch [24], making it a popular health food. The cell wall of the lotus root is composed of primary components, such as pectin, cellulose, and hemicellulose, with the accumulation and degradation of enzymes in the tissue affecting hardness. Additionally, the composition of amylose and amylopectin affects the texture [25]. Previous research also identified that crunchy lotus roots are characterized by higher water and sugar content and lower starch content, whereas mealy lotus roots show higher starch and lower moisture content [26]; our experimental results are consistent with these observations [27,28,29]. The ML varieties contain higher levels of protein, cellulose, pectin, phenolic substances, amylopectin, and cell wall materials, whereas the CL varieties have higher levels of soluble sugars, vitamin C, hemicellulose, amylose, and moisture content.

Endophytes play a crucial role in plant disease resistance and growth. In our experiments, we identified that Firmicutes, *Exiguobacterium*, and *Bradyrhizobium* were the dominant endophytic bacterial phyla and genera in ML varieties, respectively. Additionally, although Basidiomycota and Ascomycota were the dominant endophytic fungal phyla in ML and CL varieties, their proportions in ML varieties were higher than those in the CL varieties. Moreover, Firmicutes and *Exiguobacterium* were primarily observed in ML varieties, whereas *Geobacter*, *Geobacteraceae*, and *Paludibacteraceae* were the dominant endophytic bacterial genera in the CL varieties.

In contrast, Bacteroides was significantly dominant in the CL varieties. Additionally, we identified that *Exiguobacterium* was positively correlated with proteins. *Exiguobacterium* has the potential to produce cellulase [30], and *Bradyrhizobium* potentially demonstrates protein phosphorylation in both in vivo and in vitro cultures [31]. Moreover, Basidiomycota can secrete carotenoid-like pigments, lipids, phenolics, and volatile compounds [32]. Firmicutes encoded various enzymes responsible for hemicellulose and cellulose degradation, exhibiting specific roles and synergistic interactions in plant polysaccharide decomposition [33]. Bacteroides can produce cellulosomes [34], generating a free enzyme system for cellulose and hemicellulose degradation in the biomass. Ascomycota exhibit high activities of carboxymethyl cellulase and filtered paper cellulase [35].

In plants, intracellular and intercellular lipid transport is mediated by lipid-binding and transfer proteins. The ligands of these proteins form lipid molecules involved in signaling, which serve as secondary metabolites with various biological activities, thereby exhibiting diverse functions in plants [36]. The KEGG pathway results revealed that lipids were the most abundant among the differential metabolites. Additionally, hypotaurine, a reductive precursor of taurine that regulates pectin and cell metabolism pathways, exhibited higher levels of antioxidant enzyme activity and non-enzymatic antioxidant capacity, including total phenolics and vitamin C content [37]. These levels were significantly higher in the ML varieties than those in the CL varieties. Compared to the CL varieties, the ML varieties had significantly higher nucleotide metabolism pathways and a higher proportion of nucleotide transport and metabolism. Additionally, the enrichment of the taurine and hypotaurine metabolic pathways in the ML varieties was significantly higher than that in the CL varieties. The results indicated that variations in nucleotide and lipid metabolism in the ML and CL varieties contribute to their different textural qualities.

## 4. Materials and Methods

### 4.1. Field Site Description and Experimental Designs

The experiment was conducted at the Li Jian Scientific Experimental Site of the Guangxi Academy of Agricultural Sciences (108°17′ E; 23°25′ N). The soil at the experimental site was an acidic red loam with a pH of 5.49. The total nitrogen, phosphorus, and potassium levels were 0.86; 0.46; and 2.73 g·kg^−1^, respectively. Additionally, the available nitrogen, phosphorus, and potassium levels were 54.5, 8.7, and 91.0 mg·kg^−1^, respectively.

The lotus root varieties used in this study for ML properties included (a) Qintang, (b) Red beauty, and (c) Baiyupin; for CL properties they included the following: (d) Elian 6, (e) Elian 10, and (f) Dabaipong.

The plants were simultaneously sown and grown in the same field on 10 April 2023, under identical management conditions with three replications. Samples were randomly collected in November, 2023. Each sample was colonized in a 6 m^2^ library (length is 3 m and width is 2 m). In total, 20 plants were planted in each pond, and 5 plants were selected for sampling in November for each variety of lotus roots. Initially, the soil around each plant was loosened using a sterile shovel, and the plant was manually pulled out by holding the base. Subsequently, the roots were rinsed with sterile water to remove surface debris. The samples were placed in a box with ice packs and promptly transported to the laboratory. Moisture content was measured using a Nexus FT-IR spectrometer (Thermo Nicolet Corporation, Waltham, MA, USA) in the spectral range of 4000–10,000 cm [38]. The protein content of the lotus root was determined using the Kjeldahl method (Kjeldahl analyzer; Hangzhou, China), and the nitrogen conversion rate was 6.25 [39]. Soluble sugars and starch were separated using anhydrous ethanol [40].

### 4.2. Test Methods

#### 4.2.1. Analysis of Endophytic Microbial Diversity

Total DNA extraction, PCR amplification, and sequence determination of the root samples were conducted by Shanghai Majorbio Bio-pharm Technology Co., Ltd. (Shanghai, China).

Total DNA extraction was performed based on the instructions of the FastDNA^®^ Spin Kit for Rhizosphere (MP Biomedicals, Irvine, CA, USA), and DNA concentration and purity were measured using a NanoDrop 2000 spectrophotometer (Thermo Fisher Scientific, Waltham, MA, USA). PCR amplification was performed on an ABI GeneAmp^®^ 9700 system with the specific primers and sequencing types listed in Table 3.

Illumina Miseq sequencing: PCR products from the same sample were purified using the AxyPrep DNA Gel Extraction Kit (Axygen Biosciences, Union City, CA, USA), mixed, and detected through recovery using a 2% agarose gel. The recovered products were quantified using a Quantus™ fluorometer (Promega, Madison, WI, USA). Library construction used the NEXTFLEX^®^ Rapid DNA-Seq Kit.

Sequencing was performed using the Illumina MiseqPE250 and MiseqPE300 platforms (Shanghai Majorbio Bio-pharm Technology Co., Ltd., Shanghai, China). The raw data were uploaded to the NCBI database for comparison.

#### 4.2.2. Untargeted Metabolomic Assays and Analysis

A 200 μL sample was accurately transferred to a 1.5 mL centrifuge tube, followed by the addition of 800 μL of methanol and acetonitrile solution in a 1:1 ratio for extraction. After vortexing, ultrasonic extraction was performed at 5 °C and 40 kHz for 30 min. Following extraction, the sample was stored in a freezer at −20 °C for 30 min and centrifuged at 4 °C for 15 min. The supernatant was collected and dried under nitrogen atmosphere. A 1:1 mixture of acetonitrile and water was used as the compound solution, and 120 μL was collected, redissolved, and vortexed. After low-temperature ultrasonic extraction, the sample was centrifuged at 4 °C for 10 min, and the supernatant was collected and transferred to an injection vial with intubation. Ultrahigh-performance liquid chromatography (LC)-tandem Fourier transform mass spectrometry was performed using an UHPLC-QExactive system (Thermo Fisher Scientific, Waltham, MA, USA) for LC–mass spectrometry detection. Additionally, 20 μL of the supernatant was removed from each sample and used as a quality control. Chromatographic conditions were as follows: an ACQUITY UPLCHSST3 column (100 mm × 2.1 mm, i.d.1.8 μm; Waters, Milford, CT, USA) was used; mobile phase A consisted of 95% water + 5% acetonitrile + 0.1% formic acid, and mobile phase B consisted of 47.5% acetonitrile + 47.5% isopropanol + 5% water + 0.1% formic acid. The flow rate was 0.40 mL/min, injection volume was 2 μL, and column temperature was 40 °C. Multivariate analysis was conducted using the Majorbio cloud platform (https://cloud.majorbio.com, accessed on 12 November 2023).

#### 4.2.3. Statistical Analyses

The data were analyzed using Excel 2019 and SPSS 21. The results are presented as the mean ± standard deviation. Online data analysis was performed using the free online Majorbio Cloud platform (http://www.majorbio.com, accessed on 12 December 2023) of Majorbio Bio-Pharm Technology Co., Ltd. (Shanghai, China). Metabolic group data were analyzed using the Kyoto Encyclopedia of Genes and Genomes (KEGG) (www.kegg.jp/kegg/kegg1.html, accessed on12 December 2023), developed by Kanehisa Laboratories.

## 5. Conclusions

In this study, the formation mechanism of texture difference in mealy (ML) and crunchy (CL) lotus root varieties was clarified, and the differences in endophytic microbial community diversity and nucleotide as well as lipid metabolism were revealed, which were the reasons for their different textures. The functions of cellulose-degrading and protein-producing microorganisms, such as Firmicutes, Bacteroides, *Exiguobacterium*, *Bradyrhizobium*, and *Basidiomycota,* were primarily enriched in the ML varieties. Simultaneously, enhanced nucleotide transport and metabolism, together with upregulated taurine and hypotaurine pathways, contribute to the mealy texture of lotus root. In contrast, *Myxococcota*, *Geobacter*, *Paludibacteraceae*, *Rhodocyclaceae*, *Comamonadaceae*, *Micromonosporaceae*, *Sideroxydans*, *Bacillus*, *Lactococcus*, *Oxalobacteraceae*, and *Treponema* were the dominant endophytic bacterial genera in the CL varieties. The enrichment of these bacteria is one of the reasons why lotus root is crunchy.

## Figures and Tables

**Table 1:** Biological characteristics of different varieties of lotus roots.

Group	Moisture (%)	Protein (%)	Starch (mg/g)	Souble Sugar (mg/g)
ML	73.20 ± 2.65 b	1.996 ± 0.359 a	15.26 ± 2.22 a	3.00 ± 1.21 a
CL	82.26 ± 3.05 a	1.943 ± 0.106 a	8.54 ± 1.69 b	4.16 ± 0.635 a

Notes: ML—mealy lotus root; CL—crunchy lotus root. All data are presented as means ± SD (standard deviation). Groups compared using one-way ANOVA. Different letters in the same column indicate significant differences among treatments at *p* < 0.05.

**Table 2:** Significant differences of metabolites between ML and CL varieties.

Metabolite	ML/CL	M/Z	Retention Time	FDR	Regulate	Mode
Phenylpropanoids and polyketides						
2-(4-Allyl-2,6-dimethoxyphenoxy)-1-(4-Hydroxy-3-methoxyphenyl)-1-Propanol	1.39	397.16	12.07	0.0182	up	ESI+
Falcarindiol	0.72	305.17	7.57	0.0188	up	ESI−
Cavipetin C	1.21	371.25	13.19	0.0330	up	ESI+
Organoheterocyclic compounds						
Lucidenic acid J	0.57	529.21	13.19	0.0197	up	ESI+
Fragransin C1	0.80	357.16	13.23	0.1027	up	ESI+
Withaferin A	1.34	493.26	14.38	0.0135	up	ESI+
Lipids and lipid-like molecules						
Persicachrome	1.84	367.26	13.49	0.0000	up	ESI+
9,10,13-Trihydroxystearic acid	1.56	331.24	10.83	0.0001	up	ESI−
Homovanillin	1.60	167.07	13.23	0.0005	up	ESI+
Apo-12′-violaxanthal	3.50	365.24	13.25	0.0186	up	ESI+
Ganoderol A	2.16	921.70	14.42	0.0110	up	ESI−
Aflatoxin P1	1.34	340.08	5.70	0.0000	up	ESI+
(3beta,22E,24R)-5,8-Epidioxy-23-methylergosta-6,22-dien-3-ol	1.57	487.34	10.23	0.0018	up	ESI−
(3alpha,20R,24Z)-3-Hydroxy-21-oxoeupha-8,24-dien-26-oic acid	1.23	451.32	13.36	0.0008	down	ESI−
Sativan	1.30	287.12	12.50	0.0001	up	ESI+
Isofucosterol 3-O-[6-O-(9-Octadecenoyl)-b-D-glucopyranoside]	1.28	873.64	14.05	0.0007	up	ESI−
Mg(16:0/0:0/0:0)	0.70	719.56	14.32	0.0055	up	ESI−
(3beta,17alpha,23S)-17,23-Epoxy-3,28,29-trihydroxy-27-norlanost-8-en-24-one	1.22	495.31	13.34	0.0081	down	ESI−
8-Isoprostaglandin F2a	1.21	353.23	11.31	0.0004	up	ESI−
6-Ketoprostaglandin E1	0.71	333.20	8.59	0.0045	up	ESI+
Corosin	1.51	499.30	10.35	0.0092	up	ESI−
Bellidifolin	1.43	275.05	5.95	0.0036	down	ESI+
Butyryl-L-carnitine	1.40	232.15	2.48	0.0375	up	ESI+
3,7-Dimethylocta-2,6-dien-1-ol	1.21	307.26	13.84	0.0036	up	ESI−
Dihomo-alpha-linolenic acid	1.34	351.25	13.84	0.0202	up	ESI−
2-Phenylethyl 3-phenyl-2-propenoate	1.28	270.15	7.11	0.0222	down	ESI+
Taurine	1.21	124.01	0.61	0.0141	up	ESI−
2-Lysophosphatidylcholine	1.44	546.36	13.30	0.0202	up	ESI+
Prostaglandin E1	1.56	353.23	9.36	0.0550	up	ESI−
Cortexolone	1.31	347.22	10.32	0.0519	up	ESI+
Neoisoastilbin	1.29	449.11	3.82	0.0764	up	ESI−
4-oxo-Retinoic acid	1.27	315.20	9.96	0.0985	up	ESI+
Lignans, neolignans and related compounds						
1-Dihydrocarveol	1.87	350.31	14.36	0.0002	up	ESI+
Ethyl oleate	1.86	309.28	14.11	0.0017	up	ESI−
Benzenoids						
Rutacridone epoxide	1.42	304.10	6.72	0.0002	down	ESI−

Note: ML, mealy lotus; CL, crunchy lotus; ESI+, positive ion mode; ESI−, negative ion mode.

**Table 3:** Sequence type and primer sequences.

Sequencing Type	Prime Name	Prime Sequence	Length	Sequencing Platform
endopytic bacteria	799F	5′-AACMGGATTAGATACCCKG-3′	394 bp	MiseqPE250
	1193R	5′-ACGTCATCCCCACCTTCC-3′		
ITS	ITS1F	5′-CTTGGTCATTTAGAGGAAGTAA-3′	350 bp	MiSeq PE300
	ITS2F	5′-GCTGCGTTCTTCATCGATGC-3′		

## Data Availability

The raw data for soil bacterial and fungal sequencing were deposited in the NCBI Sequence Read Archive (SRA) database under accession numbers PRJNA1167891 and PRJNA1167895, respectively.
